# Modulation of perception by emotion: Altered sensitivity and perceived magnitude of negatively valenced stimuli

**DOI:** 10.3758/s13414-025-03110-w

**Published:** 2025-07-10

**Authors:** Tal Shalev, Bat-Sheva Hadad

**Affiliations:** 1 School of Neurodiversity in Education, Edmond J. Safra Brain Research Center, University of Haifa, 31905 Haifa, Israel; 2 School of Neurodiversity in Education, Edmond J. Safra Brain Research Center, University of Haifa, Haifa, Israel

**Keywords:** Emotion, Negatively valenced stimuli, Visual perception, Bayesian, Regression to the mean

## Abstract

Emotional modulation of visual processing is observed across various domains of perception. We examined whether these modulations affect perceptual sensitivity, the perceived magnitude (biases) of visual stimuli, or both. We asked participants to reproduce the duration (Exp. 1) and size (Exp. 2) of threat-related stimuli (spiders), and those of neutral ones (2D disks and butterflies). Sensitivity was examined by measuring within-subject standard deviations of reproductions for varying magnitudes of the stimuli. Biases were examined by measuring regression to the-mean, a tendency of subjective estimates to gravitate toward the center of the distribution from which stimuli were sampled. Results showed a mild increase in the standard deviations of reproductions of larger magnitudes for negatively valenced stimuli, indicating lower sensitivity. While regression biases were overall observed for these stimuli, biases decreased for the higher levels of intensities, despite their lower sensitivity. Underestimation of above-mean magnitudes was relatively moderated, demonstrating altered relations between the reliability of the sensory input and perceptual biases for these stimuli. Overall, the results suggest that magnitude perception is biased toward the central tendency of the experienced stimuli, even for threatening stimuli; however, biases are milder for the intensified values, presumably to obtain more veridical perception of these stimuli.

## Introduction

An extensive line of research has demonstrated substantial effects of emotional states and stimuli on cognitive functioning and attentional biases caused by negatively valenced stimuli. Perception is also shown to be influenced, although the nature of perceptual alterations by emotion is not completely understood. Studies have yielded inconsistent effects of negative arousal on perceptual sensitivity: discrimination of tones deteriorates when associated with an aversive stimulus (Resnik et al., 2011), but contrast sensitivity is improved when a negative stimulus acts as an attentional precue (Barbot & Carrasco, 2018). Other studies, focusing on the effects of emotions on the subjective appearance of stimuli, found that negatively arousing stimuli are perceptually biased so that, for example, spiders are rated as larger than butterflies by spider-phobic individuals (Leibovich et al., 2016). Thus, perceptual representations may be modulated by emotion by either varying their perceptual resolutions (sensitivity), their perceived magnitude (biases), or both. The current study examined the different possible alterations in perceptual representations by emotion, investigating magnitude estimations of negatively arousing and neutral stimuli.

Nonclinical, healthy individuals (Aue & Okon-Singer, 2015; but see Bar-Haim et al., 2007) and anxious individuals (Aue & Okon-Singer, 2015; Van Bockstaele at el., 2014) prioritize aversive information by enhancing attentional resources to threatening stimuli, compared with neutral ones. Spiders, snakes, and angry faces are thought to be part of a unique type of stimuli that are perceptually prioritized because of their survival importance (Brosch, et al., 2010; New et al., 2007; for review, see Sussman et al., 2016; but for different findings, see Lipp et al., 2004; LoBue, 2014; Tipples et al., 2002).

Emotion may affect other, more basic perceptual processes. There is evidence for substantial modulations for basic visual functions (neuroimaging: e.g., Morris et al., 1998; Vuilleumier, 2005; Vuilleumier et al., 2003; psychophysical: e.g., Barbot & Carrasco, 2018; Bocanegra & Zeelenberg, 2009; Ferneyhough et al., 2010; Phelps et al., 2006). The findings, however, are not conclusive (e.g., while contrast sensitivity is shown to be improved in the context of negative arousal that amplifies selective attention toward the processed stimuli; Barbot & Carrasco, 2018; Phelps et al., 2006); discrimination of tones deteriorates when it is associated with an aversive stimulus, such as odor (Resnik et al., 2011). These effects on perception may be stimulus dependent so that, for example, negative arousal impairs subsequent visual discrimination of objects but not that of faces (Mardo et al., 2018). Similarly, fearful face cues enhance orientation sensitivity for low spatial frequencies (contrast gratings) but impair sensitivity for high spatial frequencies (Bocanegra & Zeelenberg, 2009).

Critically, beyond perceptual sensitivity, emotion or negative arousal may also modulate the *veridicality* of perceptual representations. This is shown, for example, in biased representations of magnitude estimations of negatively valenced stimuli (van Ulzen et al., 2008; Whitehouse et al., 1988), and bias estimations in populations with specific phobias, in particular for feared objects such as spiders for spider-phobic individuals (Leibovich et al., 2016; Li & Graham, 2021; Shiban et al., 2016; Vasey et al., 2012). Overestimation of spiders has been shown in spider-phobic people (Li & Graham, 2021; Shiban et al., 2016), and biased size estimations are correlated with the level of fear of spiders (Vasey et al., 2012). Similarly, people with a severe fear of heights assessed balcony height as higher than people with a moderate fear of heights (Teachman et al., 2008; for a review, see Stefanucci, et al., 2011).

Subjective estimations of time are also shifted, depending on the context, emotional valence, and arousal level we experience, causing us to over- or underestimate the sense of time (for review, see Droit-Volet & Gil, 2009; Droit-Volet & Meck, 2007). Like size, time perception of negatively valenced stimuli is overestimated. Negative sounds are significantly overestimated compared with positive ones (Noulhiane et al., 2007), and the perceived duration of negative high-arousal pictures is often overestimated compared with neutral, low-arousing ones (e.g., Angrilli et al., 1997; Yamada & Kawabe, 2011). This has been attributed to the internal clock (Treisman, 1963), by which negative sounds generate a faster rate of pulses than positive sounds, resulting in subjectively longer estimations of duration (Noulhiane et al., 2007).

This line of research has focused either on perceptual resolutions (sensitivity) or subjective appearance (biases) but has hardly tested both. As perceptual biases may vary in magnitude as a function of sensory reliability and sensitivity (Petzschner et al., 2015), a comprehensive investigation of perceptual alterations by emotion should incorporate both. Furthermore, this line of research ignores substantial effects, beyond those of arousal, on perceptual estimations. The perception of magnitude, crucial to the mental representation of the physical world, is often not veridical but subject to significant contextual effects and biases. Simple judgments of magnitude are susceptible to the pervasive influence of context as strongly demonstrated, for example, by *regression to the mean*, a bias by which the perception of a given intensity of a stimulus is influenced by the overall mean of a fixed range of the sample. Intensities lower than this mean are often contracted toward the mean and thus overestimated, while those higher than the mean are, for the same reason, underestimated. This bias toward the mean reflects the most likely percept under conditions of noisy sensory input. Consequently, larger biases (regression) effects are often seen for larger magnitudes indicating the increased uncertainty for larger magnitudes and the increased standard deviations of their estimations (i.e., scalar variability; e.g., Petzschner et al., 2015).

To capture the true nature of these perceptual alterations by emotion, we examined here both the perceptual resolutions (sensitivity) and biases in the perceived magnitude of negatively arousing stimuli. We used magnitude reproduction tasks in a method of adjustment in which participants were asked to perceptually reproduce the magnitude (size or duration) of neutral or threatening stimuli, and tested regression to the mean for varying magnitudes. Perceptual estimations of negatively arousing stimuli were expected to be less reliable and thus more uncertain, but we asked whether this expected increased uncertainty would be associated with an altered magnitude of the bias. If people can exploit the uncertainty associated with measurements to optimize their responses to the statistics of the stimuli that they encounter, biases should increase in magnitude with uncertainty. If, however, a qualitatively different mechanism mediates sensitivity and magnitude perception of negatively arousing stimuli, biases would not necessarily increase with increased noise. It could be the case, for example, that overestimating the intensity of arousing stimuli may override regression to the mean such that the underestimation typically shown for higher intensities of neutral stimuli would not be evident for threatening stimuli, or generally, a more veridical magnitude perception of these stimuli would be seen.

To examine these possible mechanisms underlying perceptual modulations of negatively valenced stimuli, we measured the reproduction of arousing and neutral stimuli of varying magnitudes (duration in Exp. 1 and size in Exp. 2). We tested regression to the mean for images of spiders and butterflies, and for their controls formed of a scrambled version of each stimulus, to control for low-level visual aspects. For each magnitude, we measured the mean and the within-participant standard deviations (*SD*s) of the perceptual reproductions. The standard deviations were taken to indicate thresholds, reflecting the “area of uncertainty” for which the observer is insensitive to the difference between the magnitude of the adjusted and that of the target object (Ganel et al., 2008). The two dependent measures extracted were used to examine a) thresholds (i.e., the minimum magnitude perceived) and whether they increase proportionally with stimulus magnitude (Weber’s law) and b) the reproduction bias for each magnitude in relation to the sample mean. We also asked whether an internal emotional state of trait anxiety in the nonclinical population or a specific fear of spiders mediates these possible relations between negatively arousing stimuli and perception.

In two experiments, we employed a magnitude reproduction task in which participants were asked to perceptually reproduce the magnitude (size or duration; e.g., Cicchini et al., 2012) of neutral or threatening stimuli. In each experiment, we calculated within-subject standard deviations as an indication of sensitivity and assessed bias toward the mean (RTM). Bias was computed as the difference between the participant’s estimated scores and the physically presented values of the displayed stimulus. Regression to the mean manifests as a perceptual overestimation of values below the mean and an underestimation of values above the mean. Performance for threatening stimuli was compared with neutral (non-threatening) stimuli, and each was directly compared with its control (scrambled versions of the stimuli) to avoid any differences that might arise from irrelevant low-level visual differences between the spider and the butterfly. At the end of testing, participants were asked to complete two questionnaires using Google Forms: 1) The State-Trait Anxiety Inventory (STAI; Spielberger et al., 1983) and 2) Specific Fear of Spiders Questionnaire (FSQ; Szymanski & O’Donohue, 1995). Individual scores on the scales of trait anxiety and specific fear of spiders were regarded as continuous predictors (covariates in all analyses). Considering participants’ levels of anxiety traits, or specific fear of spiders, allowed us to investigate possible interactive effects of situational arousal and anxiety traits on visual perception. Participants were classified into two groups of relatively high (above median) and low (below median) trait anxiety or fear of spiders based on their scores in the trait section and FSQ questionnaire. Both experiments took place in our lab using a 24-in. screen, with a refresh rate of 60 Hz. Participants were seated in a quiet darken room, from a viewing distance of approximately 57 cm, using a chinrest stand to stabilize head movements. Data collection was conducted between the years 2020 and 2022, during the COVID-19 epidemic.

## Experiment 1: Duration reproduction

### Methods

#### Participants

Thirty participants (25 women, mean age = 25.17 years; range: 19–35) were included in the final analysis of the duration reproduction task.^1^ Seven additional participants were excluded from the analyses due to comparable estimations for all magnitudes presented, indicating misunderstanding or lack of attention to the task. Power analysis (using G*Power software) suggests a sample size of 30 participants is required to detect an effect size of 0.20–0.25, with an 80% power and a significant level of 0.05. Demographic data for this study was collected only for age and gender.

### Stimuli and apparatus

**Fig. 1:**
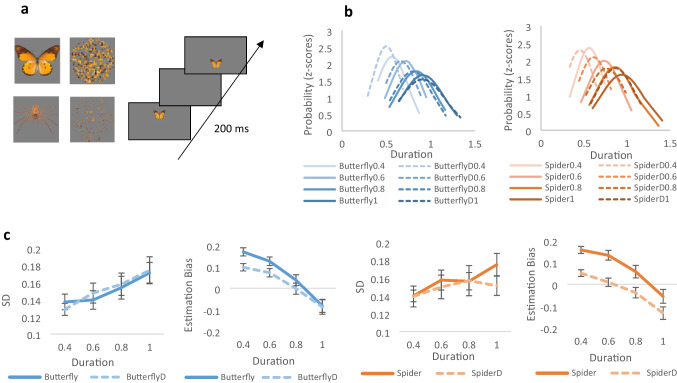
a. The stimuli used for the duration reproduction task. Upper panel: A butterfly, used as neutral stimulus, on the left, and its scrambled version (ButterflyD) on the right. Bottom panel: A light brown spider, used as a negatively valenced stimuli, on the left, and its scrambled version (SpiderD) on the right. **b.** Distribution of duration estimations (in *z* scores) for the negative and neutral stimuli presented for varying durations were computed across subjects. While estimations of durations of the butterfly and its control are contracted towards the mean, those of the spider are overestimated even for the longer-than-the-mean durations. **c** Standard deviation and mean estimation bias for the butterfly (left), and for the spider (right). Standard deviations increased linearly with increasing durations for all stimuli, consistent with Weber’s law. Despite these increasing standard deviations for longer durations, underestimations of these durations were more moderate for the spiders, indicated in the reduced estimation biases

Participants reproduced the duration of threatening or neutral stimuli. We used a single image of a spider as a negatively valenced stimulus, and of a butterfly as another living but less threatening neutral stimulus. For both, a control stimulus was composed of disks of the same size (35 mm in diameter), made of their scrambled versions (see Fig. 1a). Each stimulus type was presented in separate blocks. The presentation order was fixed using the disk versions first, followed by spiders and butterflies. The images were downloaded from the Shutterstock website and were matched in size. Each task began with a short practice of four trials. During the experiment, participants were presented with either a negatively valenced, a neutral, or a control stimulus for different time durations of 0.4, 0.6, 0.8, or 1.0 s. After the first stimuli disappeared, subjects were asked to reproduce the duration of the presented stimulus by pressing a designated key. While pressing the key, the image estimated appeared for as long as the subject estimated its duration. A total of 144 experimental trials were randomly divided into four blocks (36 repetitions for each duration) for each stimulus, with short breaks between blocks.

## Results and discussion

### Perceptual sensitivity

A mixed-design analysis of variance (ANOVA) was conducted on standard deviations with stimuli (stim vs. control) duration (0.4, 0.6, 0.8, & 1 s) and valence (negative vs. neutral) as within-subject factors. The results revealed a significant effect of duration, *F*(3,87) = 9.32, *p* < *0.0*01, η_p_^2^ = 0.24, indicating the expected increase in standard deviations with increasing intensities of the stimuli, consistent with Weber’s law. This pattern was observed for all stimulus types (Fig. 1c). There was no interaction between valence and stimuli, *F*(1,29) = 0.833, *p* = *0.3*7, η_p_^2^ = 0.028, a significant interaction was found between valence and duration, *F*(3,84) = 2.62, *p* = 0.055, η_p_^2^ = 0.083. Specific comparisons revealed marginally larger standard deviations for negatively valenced stimuli compared with their control in the longest duration (1 s), *F*(1,29) = 3.57, *p* = *0.0*69, η_p_^2^ = 0.11. No such differences were found for the neutral stimuli, *F*(1,29) = 0.101, *p* = *0.7*5, η_p_^2^ = 0.003.

### Biases

To test for modulations in the perceived magnitude of negative stimuli, a mixed-design ANOVA was carried out on the estimation bias (difference in the estimations and the physical duration of the displayed stimulus, calculated as estimate scores minus physical size), with stimuli (stimulus vs. control), duration (0.4, 0.6, 0.8, & 1.0 s), and valence (negative vs. neutral) as within-subject factors. The analysis revealed a significant interaction between stimuli and duration, *F*(3,87) = 4.81, *p* = 0.004, η_p_^2^ = 0.14, and between stimuli and valence, *F*(1,29) = 7.43, *p* = 0.011, η_p_^2^ = 0.204. These interactions indicated that while estimations of the butterfly and its control were similarly contracted toward the mean *F*(1,29) = 1.19, *p* = 0.29, η_p_^2^ = 0.03, those of the spider were overestimated compared with its control, *F*(1,29) = 14.99, *p* < *0.0*01, η_p_^2^ = 0.34. Underestimations of the longer-than-the-mean durations were specifically smaller for the spiders, even for the longest durations, *F*(1,29) = 6.74, *p* = *0.0*15, η_p_^2^ = 0.19. Thus, compared with those of the control stimulus, the durations of the spider were perceptually overestimated, indicating an altered subjective appearance of negatively arousing stimuli (see Fig. 1). Interestingly, despite the somewhat increased SDs for increasing magnitudes, smaller biases were seen for of the negatively valenced stimulus.

A mixed-design ANOVA, carried out with trait anxiety levels (based on STAI scores) as a between-subject factor, revealed no significant interactions between trait anxiety levels and the other factors, indicating that this pattern of increased overestimation of the negatively arousing stimuli was observed for both high and low anxiety traits.

In conclusion, standard deviation scores, reflecting perceptual sensitivity, generally increased for all stimulus types, consistent with Weber’s law. However, marginally larger standard deviations were found for negatively valenced stimuli compared with their control at longer durations, whereas neutral stimuli did not differ from their control in any of the tested conditions. The examination of estimation bias in perceived magnitude revealed a similar pattern of contraction toward the mean for the butterfly and its control, but significantly smaller biases for the spider compared with its control, despite somewhat lower sensitivity.

## Experiment 2: Size reproduction

### Methods

#### Participants

Twenty-two individuals (19 women, mean age = 26.8 years, range: 18–40) participated in the size reproduction task for course credits or payment. All participants had normal or corrected-to-normal vision, with no known neurological disorders, ADHD, learning disabilities, or psychiatric disorders. The experiment was approved by the ethics committee of the University of Haifa. Participants signed an informed consent form.

#### Stimuli and apparatus

**Fig. 2:**
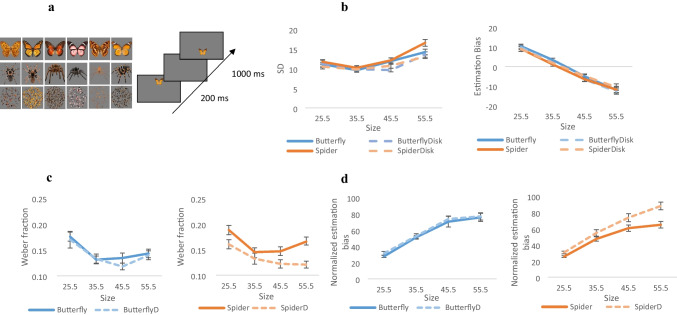
**a** Left: The stimuli used in the size reproduction task. Upper panel: Butterflies, used for neutral stimuli. Middle: ecological spiders, used as negatively valenced stimuli. Lower panel: Scrambled version of each stimulus used as control stimuli. Right: Examples of a display used in the size reproduction task. Participants were asked to reproduce the size of the upper butterfly by adjusting the butterfly at the bottom. The adjusted butterfly was presented after a 1-s delay from the initial presentation. Butterflies, spiders, and disks were presented on separate blocks. **b** Standard deviation and mean estimation bias for all stimuli. *SD*s increased linearly with increasing size, consistent with Weber’s law. No difference was demonstrated for the estimation bias. **c** Weber fractions (*SD*/estimation) differed between the spider and the control stimuli with significantly higher Weber fractions observed for the spider. **d** Normalized estimation bias for the Butterfly and its control (ButterflyD, left), and for the spider and its control (SpiderD, right) in the size estimation task. Results show significantly smaller biases for the spiders compared to control stimuli, for larger magnitudes

In this task, we expanded the stimulus set to avoid potential adaptation effects. Six different images of spiders were used as negatively valenced stimuli and six images of butterflies as another living but less threatening neutral stimuli. For both spiders and butterflies, control stimuli were composed of a total of six disks of the same size, made up of their scrambled versions—three for each one (see Fig. 2a). The different stimulus types were presented in separate blocks, with a fixed presentation order: disk versions first, followed by spiders and butterflies. Each task began with a short practice of four trials. A total of 72 experimental trials were randomly divided into four blocks (18 repetitions for each size), with short breaks between blocks, for each stimulus.

The target was displayed in either 25.5, 35.5, 45.5, or 55.5 mm diameter size on a gray background. The stimulus was displayed 3 cm above the center for 200 ms, followed by a 1,000-ms delay. Then, an adjustable stimulus appeared, positioned at 3 cm below the center (see Fig. 2a), one of either two sizes—20 or 61 mm diameter size. Participants were asked to fit the second, adjustable, stimulus to the first, using the up and down arrow keys.

### Results and discussion

#### Perceptual sensitivity

A mixed-design ANOVA was conducted on standard deviations of size estimations, with stimuli (stim vs. control) size (25.5, 35.5, 45.5, or 55.5 mm) and valence (negative vs. neutral) as within-subject factors. Results revealed a significant three-way interaction between stimuli, valence, and size, *F*(3,63) = 2.85, *p* = 0.044, η_p_^2^ = 0.12. This interaction indicates increased standard deviation values specifically for the negative stimuli compared with its control, *F*(1,21) = 15.06, *p* < *0.0*01, η_p_^2^ = 0.42, and for larger magnitudes in particular, *F*(1,21) = 19.72, *p* < *0.0*01, η_p_^2^ = 0.48 (see Fig. 2b). The same pattern was shown for the Weber fractions (computed as mean standard deviation divided by the physical size): a significant effect of size, *F*(3,63) = 32.48, *p* < *0.0*01, η_p_^2^ = 0.60, and a significant interaction between stimuli and valence, *F*(1,21) = 5.98, *p* = 0.023, η_p_^2^ = 0.22, indicated higher Weber fractions for the spider compared with its controls; for both the 2D disk, *F*(1,21) = 16.55, *p* < *0.0*01, η_p_^2^ = 0.60 and for the butterfly, *F*(1,21) = 5.89, *p* = 0.024, η_p_^2^ = 0.22 (see Fig. 2c).

#### Biases

As in the duration experiment, the analysis was carried out on the estimation bias (estimated minus physical size), computed for each size. A mixed-design ANOVA revealed a significant interaction between stimuli and valence, *F*(1,21) = 5.15, *p* = 0.034, η_p_^2^ = 0.197, and between valence and size, *F* (3,63) = 4.03, *p* = 0.011, η_p_^2^ = 0.16 (see Fig. 2b). However, because of significant differences in standard deviations between stimuli, we normalized estimation biases by dividing the mean estimation by the Weber fractions. The analysis revealed a significant three-way interaction between stimuli, valence, and size, *F*(3,63) = 2.85, *p* = 0.044, η_p_^2^ = 0.12, indicating smaller biases for the spider stimuli compared with its control, *F*(3,63) = 4.10, *p* = 0.010, η_p_^2^ = 0.16 (see Fig. 2d). A specific comparison between the butterfly and the spider further revealed a marginal difference between the two stimuli for the larger magnitude (55.5 mm), *F*(1,21) = 4.00, *p* = 0.059, η_p_^2^ = 0.16. 

**Fig. 3:**
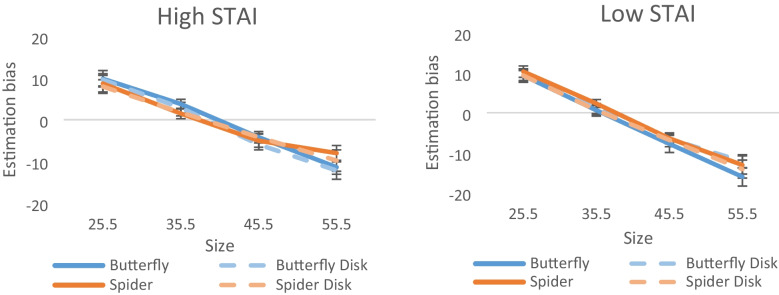
Estimation bias for high- and low-anxiety trait groups. Moderate underestimation was observed for the spider compared to the control and neutral stimuli, particularly in the high-STAI-score group

A mixed-design ANOVA carried out with trait anxiety levels (based on STAI scores) as a between-subject factor revealed a significant interaction between valence, size, and STAI group, *F*(3,60) = 3.31, *p* = 0.026, η_p_^2^ = 0.142. This interaction demonstrates that the underestimation of the larger magnitudes (55.5 mm) was more subtle for the negative stimuli in particular for those with relatively high levels of anxiety trait (see Fig. 3).

The examination of perceptual sensitivity in size estimation revealed an interesting pattern of results, demonstrating significantly lower sensitivity (higher Weber fractions) for the spider compared with both its scrambled control and the butterfly. Despite these differences in perceptual sensitivity, we did not find an increase in estimation bias for the spider compared with its control. On the contrary, biases were significantly smaller. This pattern of results suggests altered principles of Bayesian inference in processing such stimuli, where biases do not appear to increase to compensate for reduced sensitivity.

Moreover, we found a more subtle underestimation of larger magnitudes for the spider in participants with relatively higher anxiety traits. This finding provides further support for a possible mechanism underlying the altered biases observed in the overall sample: milder underestimation biases for intense magnitudes of negatively arousing stimuli may allow sensory regulation mechanisms while also preserving a more veridical perception of the threatening stimulation.

## General discussion

We investigated perceptual alterations by emotion examining both resolutions (sensitivity) of perceptual representations and their subjective appearance (biases). For both duration and size estimations, we tested regression to the mean—a tendency of subjective estimates to be biased toward the center of the distribution. As perceptual biases and their magnitude may vary as a function of sensory reliability and sensitivity (Petzschner et al., 2015), we also examined whether the expected increase of the regression effect for larger and noisier magnitudes would be modulated in the context of negative arousal. Importantly, the analysis focused on the differences between each stimulus (spider or butterfly) and its scrambled control to ensure that any observed differences between the butterfly and the spider can be attributed to arousal rather than potential variations in low-level visual features. Individual differences were also examined using the STAI and the FSQ.

We found mild modulations both in the perceptual sensitivity and in the pattern of regression-to-the-mean for the negatively valenced stimuli, in both duration and size estimation tasks. Although thresholds were elevated compared with neutral stimuli, a reduced bias was obtained for larger magnitudes in both tasks. This pattern violates Bayesian principles by which prior knowledge and biases increasingly affect perception with increasing uncertainty. For neutral stimuli, estimations of larger magnitudes exhibited increasing standard deviations (i.e., scalar variability, e.g., Petzschner et al., 2015) and were indeed biased more than smaller magnitudes.

The somewhat elevated thresholds in larger magnitudes, indicating reduced sensitivity to differences in the magnitude of negatively valenced stimuli, are consistent with previous findings demonstrating poorer sensitivity in discriminating stimuli conditioned with an aversive stimulus (Resnik et al., 2011), or stimuli presented immediately following an aversive stimulus (Mardo et al., 2018). This modulated sensitivity indicates that although attentional biases and processing priorities are given for negative information at the expense of other, lower-priority stimuli (Mather & Sutherland, 2011), discrimination of these stimuli is poorer and has lower resolutions in detecting subtle differences in certain conditions. Deterioration in performance was also found in other studies of perceptual discrimination tasks using aversive versus neutral/positive conditioning or aversive outcomes (Laufer & Paz, 2012; Laufer et al., 2016; Manassero et al., 2019; Schechtman et al., 2010; Shalev et al., 2018). For example, after a conditioning session where aversive information (images) was paired with neutral stimuli (auditory or visual), participants exhibited increased thresholds (Shalev et al., 2018). The authors suggested that these changes in sensitivity occurred due to perceptual learning and circuit plasticity in sensory discrimination—implicating perhaps a central mechanism that may be used during learning to modulate early sensory neural representations (Shalev et al., 2018). Activation in the amygdala, insula, early visual cortex, and anterior cingulate cortex was attributed to the increase in thresholds after aversive compared with nonaversive learning (Laufer & Paz, 2012; Laufer et al., 2016; Shalev et al., 2018). Decreased sensitivity in the discrimination of negatively valenced stimuli has also been attributed to wider behavioral generalization, thus facilitating faster, more defensive reactions, avoiding slight differences between stimuli (Resnik et al., 2011; Schechtman et al., 2010; Shalev et al., 2018). Either way, it may be that in the presence of threat or danger, one must respond quickly, taking into account that he might be wrong. Making a mistake, for that matter, would have little impact compared with the parallel scenario of being right. In this “better safe than sorry” approach, biases toward wider generalization are expected and lead in turn to decreased sensitivity (Laufer & Paz, 2012).

The same mechanism suggested for the decreased sensitivity in estimating the magnitude of negatively valenced stimuli may also underlie the modulated biases. The reduced bias for larger, noisier magnitudes of negative stimuli may serve the same approach of keeping as much information about important features of these stimuli as possible. Although this does not adhere to Bayesian principles of increased bias effects with decreasing reliability of sensory input (e.g., Petzschner et al., 2015), it provides important means by which the perceptual system deals with negative valence. The need to reach a veridical subjective appearance of negative stimuli and their basic features (e.g., size, duration) may override the general need of the system to reduce noise. The system thus specifically restrains the underestimation of negative stimuli of relatively large magnitudes, presumably to prevent it from missing increasing magnitudes of threatening stimuli.

Although this pattern of results was shown for both dimensions, the size reproduction task elicited larger differences in sensitivity between negative and neutral stimulus compared with duration. The stronger difference in the pattern of results found for the size compared with the duration estimation experiment, most likely relates to the known specialization of the visual system in processing spatial information (i.e., size), compared with processing temporal information (i.e., duration). Because the task involves a reproduction of visual stimuli, duration judgments were indeed much noisier than size (e.g., Binur & Hadad, 2023). Consequently, as predicated by Bayesian models of perception, accumulated statistics serve as prior knowledge and more robustly biased estimations of durations. Importantly, however, modulated biases for negatively arousing stimuli were also seen for size when estimation biases were normalized to the relatively higher standard deviations observed for the negative stimuli. Because Weber fractions were found significantly higher for the negative compared with the neutral stimuli in the size reproduction task, estimation biases were normalized, revealing lower biases for the spider compared with its control.

Specifically, in the case of size estimations, the modulations of the bias vary for people with high and low anxiety trait. Although the pattern of results was similar for both groups, the reduced underestimation for larger magnitudes (45.5 mm, 55.5 mm) in the negatively valenced stimuli was more robust for the high anxiety trait group. Given that people with relatively high anxiety in the current study are healthy individuals with no clinical diagnosis of anxiety disorders, the results of the present study suggest a general typical mechanism by which the perceptual system processes threat-related stimuli. The modulated biases seen across the two groups for durations reproductions, the more uncertain measurements strongly support this conclusion. Note, however, that contrary to the differences observed between individuals in the general anxiety traits, no substantial differences were found for fear of spiders. Only four participants reported high levels of fear of spiders and thus analysis was carried out across all levels. Yet the current results, demonstrating mild perceptual modulations by emotion for the general nonclinical populations with no specific fear of spiders, suggest a general mechanism of emotion–perception interactions in the healthy perceptual system. A different mechanism may operate in those clinically diagnosed with anxiety, and further research on clinical populations can shed light on emotion–perception interactions in this perhaps less adaptive perceptual system.

We show here that perceptual modulations by emotion occur both by mildly varying the resolutions (sensitivity) of perceptual representations and by slightly modifying their subjective appearance (biases). Regression biases are observed for all stimuli, including the negatively valenced stimuli. These results are consistent with recent findings demonstrating intact adaptation effects, suggesting that basic calibration mechanisms of magnitude perception also function of negative stimuli (Shalev & Hadad, 2025). However, there are some modulations of the subjective appearance of negatively valenced stimuli, specifically manifested in moderated underestimations of the above-mean magnitudes of these stimuli. These subtle alterations in perceptual representations by emotion, presumably interfering with Bayesian computations aiming at reducing the noise of perceptual representations, restraining the underestimation of negative stimuli to obtain a veridical impression of increasing magnitudes of threatening stimuli.

## Data Availability

All data analyzed during the current study will be made publicly available at the Open Science Framework (OSF) upon publication. 10.17605/OSF.IO/8CSG5

## Footnotes

1 One participant used a hearing aid in one ear. The results did not change with and without his data included.

## Code availability

All code used in the current study will be made publicly available at the Open Science Framework (OSF) upon publication. 10.17605/OSF.IO/8CSG5

## References

[CR1] Angrilli, A., Cherubini, P., Pavese, A., & Manfredini, S. (1997). The influence of affective factors on time perception. *Perception & Psychophysics,**59*(6), 972–982. 9270369 10.3758/bf03205512

[CR2] Aue, T., & Okon-Singer, H. (2015). Expectancy biases in fear and anxiety and their link to biases in attention. *Clinical Psychology Review,**42*, 83–95. 26379081 10.1016/j.cpr.2015.08.005

[CR3] Barbot, A., & Carrasco, M. (2018). Emotion and anxiety potentiate the way attention alters visual appearance. *Scientific Reports*, *8*(1), Article 5938.‏ 10.1038/s41598-018-23686-8 10.1038/s41598-018-23686-8 PMC5897558 29651048

[CR4] Bar-Haim, Y., Lamy, D., Pergamin, L., Bakermans-Kranenburg, M. J., & Van Ijzendoorn, M. H. (2007). Threat-related attentional bias in anxious and nonanxious individuals: A meta-analytic study. *Psychological Bulletin,**133*(1), 1–24. 17201568 10.1037/0033-2909.133.1.1

[CR5] Binur, N., & Hadad, B.-S. (2023). Contextual effects on duration perception are modality-specific. *Journal of Experimental Psychology: Human Perception and Performance,**49*(11), 1420–1429. 10.1037/xhp0001164 37870821 10.1037/xhp0001164

[CR6] Bocanegra, B. R., & Zeelenberg, R. (2009). Emotion improves and impairs early vision. *Psychological Science,**20*(6), 707–713. 19422624 10.1111/j.1467-9280.2009.02354.x

[CR7] Brosch, T., Pourtois, G., & Sander, D. (2010). The perception and categorisation of emotional stimuli: A review. *Cognition and Emotion,**24*(3), 76–108.

[CR8] Cicchini, G. M., Arrighi, R., Cecchetti, L., Giusti, M., & Burr, D. C. (2012). Optimal encoding of interval timing in expert percussionists. *Journal of Neuroscience, 32*(3), 1056–1060. 10.1523/JNEUROSCI.3411-11.2012 PMC6621155 22262903

[CR9] Droit-Volet, S., & Gil, S. (2009). The time–emotion paradox. *Philosophical Transactions of the Royal Society B: Biological Sciences,**364*(1525), 1943–1953. 10.1098/rstb.2009.0013 PMC2685815 19487196

[CR10] Droit-Volet, S., & Meck, W. H. (2007). How emotions colour our perception of time. *Trends in Cognitive Sciences,**11*(12), 504–513. 18023604 10.1016/j.tics.2007.09.008

[CR11] Ferneyhough, E., Stanley, D. A., Phelps, E. A., & Carrasco, M. (2010). Cuing effects of faces are dependent on handedness and visual field. *Psychonomic Bulletin & Review,**17*, 529–535. 20702873 10.3758/PBR.17.4.529 PMC3150162

[CR12] Ganel, T., Chajut, E., & Algom, D. (2008). Visual coding for action violates fundamental psychophysical principles. *Current Biology,**18*(14), R599–R601. 18644333 10.1016/j.cub.2008.04.052

[CR13] Laufer, O., Israeli, D., & Paz, R. (2016). Behavioral and neural mechanisms of overgeneralization in anxiety. *Current Biology,**26*(6), 713–722. 26948881 10.1016/j.cub.2016.01.023

[CR14] Laufer, O., & Paz, R. (2012). Monetary loss alters perceptual thresholds and compromises future decisions via amygdala and prefrontal networks. *Journal of Neuroscience,**32*(18), 6304–6311. 22553036 10.1523/JNEUROSCI.6281-11.2012 PMC6622137

[CR15] Leibovich, T., Cohen, N., & Henik, A. (2016). Itsy bitsy spider? Valence and self-relevance predict size perception. *Biological Psychology, 121*(Pt. B), 138–145. 10.1016/j.biopsycho.2016.01.009 10.1016/j.biopsycho.2016.01.009 PMC5154329 26802365

[CR16] Li, S. H., & Graham, B. M. (2021). Mind’s eye: The impact of spider presence and cognitive therapy on size estimation biases in spider phobia. *Journal of Anxiety Disorders, 83,* Article 102456.‏ 10.1016/j.janxdis.2021.102456 34340170

[CR17] Lipp, O. V., Derakshan, N., Waters, A. M., & Logies, S. (2004). Snakes and cats in the flower bed: Fast detection is not specific to pictures of fear relevant animals. *Emotion,**4*, 233–250. 10.1037/1528-3542.4.3.233 15456393 10.1037/1528-3542.4.3.233

[CR18] LoBue, V. (2014). Deconstructing the snake: The relative roles of perception, cognition and emotion on threat detection. *Emotion,**14*, 701–711. 10.1037/a0035898 24708497 10.1037/a0035898

[CR19] Manassero, E., Mana, L., Concina, G., Renna, A., & Sacchetti, B. (2019). Implicit and explicit systems differently predict possible dangers. *Scientific Reports,**9*(1), 1–12. 31527740 10.1038/s41598-019-49751-4 PMC6746769

[CR20] Mather, M., & Sutherland, M. R. (2011). Arousal-biased competition in perception and memory. *Perspectives on Psychological Science,**6*(2), 114–133. 10.1177/1745691611400234 21660127 10.1177/1745691611400234 PMC3110019

[CR21] Mardo, E., Schwartz, S., Avidan, G., & Hadad, B. S. (2018). Emotional cues differently modulate visual processing of faces and objects. *Emotion*. 10.1037/emo0000453 10.1037/emo0000453 29878801

[CR22] Morris, J. S., Friston, K. J., Büchel, C., Frith, C. D., Young, A. W., Calder, A. J., & Dolan, R. J. (1998). A neuromodulatory role for the human amygdala in processing emotional facial expressions. *Brain: A Journal of Neurology*, *121*(1), 47–57.‏ 10.1093/brain/121.1.47 9549487

[CR23] New, J., Cosmides, L., & Tooby, J. (2007). Category-specific attention for animals reflects ancestral priorities, not expertise. *Proceedings of the National Academy of Sciences,**104*(42), 16598–16603. 10.1073/pnas.0703913104 PMC2034212 17909181

[CR24] Noulhiane, M., Mella, N., Samson, S., Ragot, R., & Pouthas, V. (2007). How emotional auditory stimuli modulate time perception. *Emotion,**7*(4), 697–704. 18039036 10.1037/1528-3542.7.4.697

[CR25] Petzschner, F. H., Glasauer, S., & Stephan, K. E. (2015). A Bayesian perspective on magnitude estimation. *Trends in Cognitive Sciences,**19*(5), 285–293. 25843543 10.1016/j.tics.2015.03.002

[CR26] Phelps, E. A., Ling, S., & Carrasco, M. (2006). Emotion facilitates perception and potentiates the perceptual benefits of attention. *Psychological Science,**17*(4), 292–299. 16623685 10.1111/j.1467-9280.2006.01701.x PMC1555625

[CR27] Resnik, J., Sobel, N., & Paz, R. (2011). Auditory aversive learning increases discrimination thresholds. *Nature Neuroscience,**14*(6), 791–796. 21552275 10.1038/nn.2802

[CR28] Schechtman, E., Laufer, O., & Paz, R. (2010). Negative valence widens generalization of learning. *Journal of Neuroscience,**30*(31), 10460–10464. 20685988 10.1523/JNEUROSCI.2377-10.2010 PMC6634660

[CR29] Shalev, L., Paz, R., & Avidan, G. (2018). Visual aversive learning compromises sensory discrimination. *Journal of Neuroscience,**38*(11), 2766–2779. 29439168 10.1523/JNEUROSCI.0889-17.2017 PMC6595998

[CR30] Shalev, T., Hadad, B. S. (2025). Reduced sensitivity but intact adaptation effects in magnitude perception of negative stimuli. Submitted.

[CR31] Shiban, Y., Fruth, M. B., Pauli, P., Kinateder, M., Reichenberger, J., & Mühlberger, A. (2016). Treatment effect on biases in size estimation in spider phobia. *Biological Psychology,**121*, 146–152. 26987423 10.1016/j.biopsycho.2016.03.005

[CR32] Spielberger, C. D., Gorsuch, R. L., Lushene, R., Vagg, P. R., & Jacobs, G. A. (1983). *Manual for the state-trait anxiety inventory*. Consulting Psychologists Press.

[CR33] Stefanucci, J. K., Gagnon, K. T., & Lessard, D. A. (2011). Follow your heart: Emotion adaptively influences perception. *Social and Personality Psychology Compass,**5*(6), 296–308. 10.1111/j.17519004.2011.00352 21731579 10.1111/j.1751-9004.2011.00352.x PMC3124782

[CR34] Sussman, T. J., Jin, J., & Mohanty, A. (2016). Top-down and bottom-up factors in threat-related perception and attention in anxiety. *Biological Psychology,**121*, 160–172. 27546616 10.1016/j.biopsycho.2016.08.006

[CR35] Szymanski, J., & O’Donohue, W. (1995). Fear of spiders questionnaire. *Journal of Behavior Therapy and Experimental Psychiatry,**26*(1), 31–34. 7642758 10.1016/0005-7916(94)00072-t

[CR36] Teachman, B. A., Stefanucci, J. K., Clerkin, E. M., Cody, M. W., & Proffitt, D. R. (2008). A new mode of fear expression: Perceptual bias in height fear. *Emotion,**8*(2), 296–301. 10.1037/1528-3542.8.2.296 18410203 10.1037/1528-3542.8.2.296 PMC3182306

[CR37] Tipples, J., Young, A. W., Quinlan, P., Broks, P., & Ellis, A. W. (2002). Searching for threat. *The Quarterly Journal of Experimental Psychology Section A,**55*(3), 1007–1026. 10.1080/02724980143000659 10.1080/02724980143000659 12188507

[CR38] Treisman, M. (1963). Temporal discrimination and the indifference interval: Implications for a model of the “internal clock.” *Psychological Monographs: General and Applied,**77*(13), 1–31. 10.1037/h0093864 5877542

[CR39] Van Bockstaele, B., Verschuere, B., Tibboel, H., De Houwer, J., Crombez, G., & Koster, E. H. (2014). A review of current evidence for the causal impact of attentional bias on fear and anxiety. *Psychological Bulletin,**140*(3), 682–721. 24188418 10.1037/a0034834

[CR40] van Ulzen, N. R., Semin, G. R., Oudejans, R. R., & Beek, P. J. (2008). Affective stimulus properties influence size perception and the Ebbinghaus illusion. *Psychological Research Psychologische Forschung,**72*, 304–310. 10.1007/s00426-007-0114-6 17410379 10.1007/s00426-007-0114-6 PMC2668624

[CR41] Vasey, M. W., Vilensky, M. R., Heath, J. H., Harbaugh, C. N., Buffington, A. G., & Fazio, R. H. (2012). It was as big as my head, I swear!: Biased spider size estimation in spider phobia. *Journal of Anxiety Disorders,**26*(1), 20–24. 10.1016/j.janxdis.2011.08.00 21906909 10.1016/j.janxdis.2011.08.009 PMC3244514

[CR42] Vuilleumier, P. (2005). How brains beware: Neural mechanisms of emotional attention. *Trends in Cognitive Sciences,**9*(12), 585–594. 16289871 10.1016/j.tics.2005.10.011

[CR43] Vuilleumier, P., Armony, J. L., Driver, J., & Dolan, R. J. (2003). Distinct spatial frequency sensitivities for processing faces and emotional expressions. *Nature Neuroscience,**6*(6), 624–631. 12740580 10.1038/nn1057

[CR44] Whitehouse, A. M., Freeman, C. P., & Annandale, A. (1988). Body size estimation in anorexia nervosa. *The British Journal of Psychiatry,**153*(2), 23–26. 3242746

[CR45] Yamada, Y., & Kawabe, T. (2011). Emotion colors time perception unconsciously. *Consciousness and Cognition,**20*(4), 1835–1841. 21764331 10.1016/j.concog.2011.06.016

